# Does *Salmonella diarizonae* 58:r:z_53_ Isolated from a Mallard Duck Pose a Threat to Human Health?

**DOI:** 10.3390/ijms25115664

**Published:** 2024-05-23

**Authors:** Karolina Wódz, Lidia Piechowicz, Ewa Tokarska-Pietrzak, Jan Gawor, Robert Gromadka, Zbigniew Bełkot, Zuzanna Strzałkowska, Jan Wiśniewski, Tomasz Nowak, Janusz Bogdan, Krzysztof Anusz, Joanna Pławińska-Czarnak

**Affiliations:** 1 Laboratory of Molecular Biology, Vet-Lab Brudzew, 62-720 Brudzew, Poland; tomasz@labbrudzew.pl; 2 Department of Medical Microbiology, Faculty of Medicine, Medical University of Gdańsk, 80-204 Gdańsk, Poland; lidiap@gumed.edu.pl (L.P.); etok@gumed.edu.pl (E.T.-P.); 3 DNA Sequencing and Synthesis Facility, Institute of Biochemistry and Biophysics, Polish Academy of Sciences, 02-106 Warsaw, Poland; gaworj@wp.pl (J.G.); robert@ibb.waw.pl (R.G.); 4 Department of Food Hygiene of Animal Origin, Faculty of Veterinary Medicine, University of Life Sciences in Lublin, 20-950 Lublin, Poland; zbigniew.belkot@up.lublin.pl; 5 Department of Food Hygiene and Public Health Protection, Institute of Veterinary Medicine, Warsaw University of Life Sciences, 02-776 Warsaw, Poland; z.strzalkowska@gmail.com (Z.S.); jan_wisniewski1@sggw.edu.pl (J.W.); janusz_bogdan@sggw.edu.pl (J.B.); krzysztof_anusz@sggw.edu.pl (K.A.)

**Keywords:** *Salmonella diarizonae* 58:r:z_53_, wild duck, AMR, plasmid, complete genome, sequencing, virulence genes, type-III effectors, public health

## Abstract

*Salmonella diarizonae* (IIIb) is frequently isolated from reptiles and less frequently from birds and mammals. However, its isolation from invasive human infections has not been widely reported. Migratory mallard ducks are excellent bioindicators of pathogen presence and pathogen antibiotic resistance (AMR). We present the first isolation from a mallard duck in central Europe of the antibiotic-resistant *Salmonella enterica* subsp. *diarizonae* with the unique antigenic pattern 58:r:z_53_ and report its whole-genome sequencing, serosequencing, and genotyping, which enabled the prediction of its pathogenicity and comparison with phenotypic AMR. The isolated strain was highly similar to *S. diarizonae* isolated from humans and food. Twenty-four AMR genes were detected, including those encoding aminoglycoside, fluoroquinolone, macrolide, carbapenem, tetracycline, cephalosporin, nitroimidazole, peptide antibiotic, and disinfecting agent/antiseptic resistance. Six *Salmonella* pathogenicity islands were found (SPI-1, SPI-2, SPI-3, SPI-5, SPI-9, and SPI-13). An iron transport system was detected in SPI-1 centisome C63PI. Plasmid profile analyses showed three to be present. Sequence mutations in the *invA* and *invF* genes were noted, which truncated and elongated the proteins, respectively. The strain also harbored genes encoding type-III secretion-system effector proteins and many virulence factors found in *S. diarizonae* associated with human infections. This study aims to elucidate the AMR and virulence genes in *S. enterica* subsp. *diarizonae* that may most seriously threaten human health.

## 1. Introduction

The *Salmonella* genus, containing 2659 serovars, is divided into two species: *Salmonella enterica* with 2637 and *Salmonella bongori* with the remaining 22. There are six subspecies of *Salmonella enterica*: *enterica* (I) containing 1586 serovars, *salamae* (II) with 522, *arizonae* (IIIa) with 102, *diarizonae* (IIIb) with 338, *houtenae* (IV) with 76, and *indica* (VI) with the final 13 [1]. The *Salmonella enterica* species is a widely known pathogen causing serious illness in humans and animals. Some serotypes of *Salmonella enterica* subsp. *enterica* are particularly well described and are known to cause a wide range of food- and water-borne illnesses [2,3,4,5,6,7]. *Salmonella enterica* subsp. *diarizonae* is the third most abundant subspecies of the genus. The *diarizonae* (IIIb) subspecies is commonly isolated from a variety of cold-blooded animals, including snakes, turtles, and lizards, as well as from birds, sheep, and even humans [8,9,10,11]. Although human infection caused by *S. diarizonae* seems to be rare, more and more cases of gastroenteritis and bacteremia are being described in the global literature [12,13,14].

*Salmonella diarizonae* 58:r:z_53_ was first isolated in Germany in 1985 from snake feces and in 2021 it was isolated in Poland as a multidrug-resistant bacterium from a mallard duck (*Anas platyrhynchos*). In this study, we present a genome analysis of this rare multidrug-resistant strain to demonstrate its potential human pathogenicity and virulence features.

## 2. Results

### 2.1. Source Animal and Antimicrobial Resistance of the Isolate

The mallard duck from the intestine of which the *S. enterica* subsp. *diarizonae* 58:r:z_53_ (*S*. IIIb 58:r:z_53_) strain was isolated had no pathological changes when given an anatomopathological examination and was in good physical condition. The isolated strain was found to be resistant to 14 of the 33 antibiotics tested [15]. It was sensitive to ampicillin, amoxicillin, trimethoprim-sulfamethoxazole, third-generation cephalosporin, and imipenem.

### 2.2. Phylogenetic Analysis of Salmonella enterica subsp. diarizonae IIIb 58:r:z_53_

**Figure 1:**
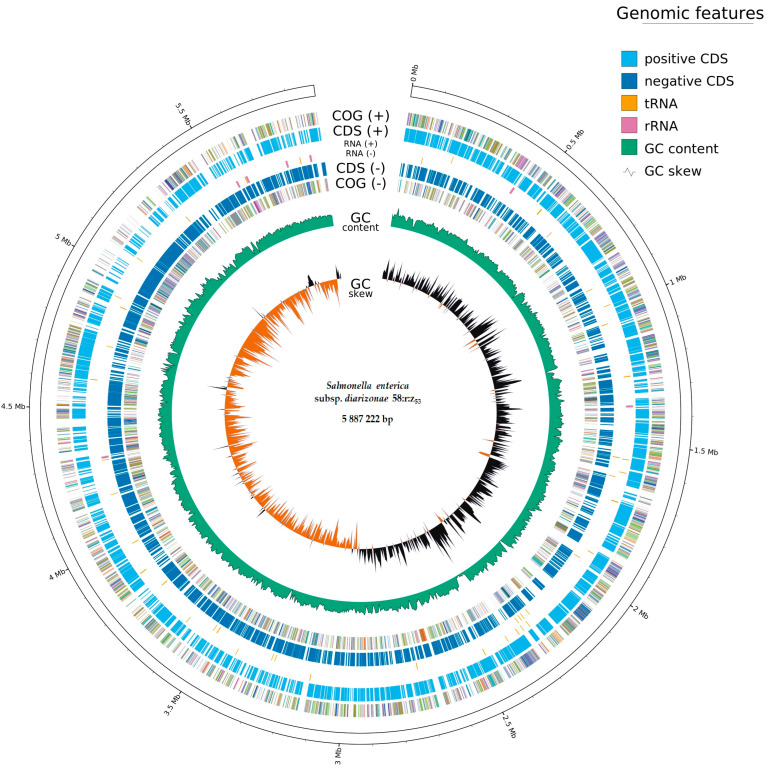
Illustration of the distribution of the genome annotations of *Salmonella enterica* subsp. *diarizonae* 58:r:z_53_ isolated from a mallard duck in Poland. The protein-coding sequence (CDS) elements of the figure are shown in gray for the position label (Mbp: megabase pairs); GC: guanine and cytosine; tRNA: transfer RNA; rRNA: ribosomal RNA. Visualized with GenoVi [16], accessed on 6 March 2024.

**Figure 2:**
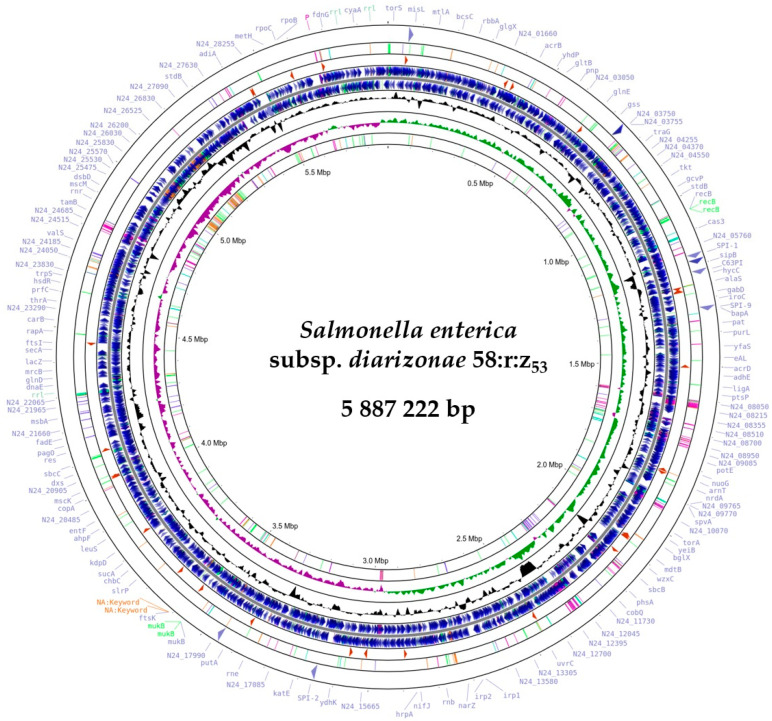
Genome map of *S. enterica* subsp. *diarizonae* 58:r:z_53_. The innermost rings show genome positions (Mbp: megabase pairs) and GC content, shown in black. The outer rings represent the coding orientation, with the forward strand on the outside and the reverse strand on the inside. Created with Proksee [17].

The assembly of sequence reads revealed a single circular chromosome with a total length of 5,887,222 bp and an average guanine–cytosine content of 51.32%. This genome had 6308 protein-coding sequences (CDS), 102 transfer RNA (tRNA) genes, and 22 ribosomal RNA (rRNA) genes (Figure 1), Whole Genome map of *S. enterica* subsp. *diarizonae* 58:r:z_53_ (Figure 2).

### 2.3. Genotypic Serotyping

Multidirectional whole-genome sequencing (WGS) analysis predicted the O antigen to be the serotype 58 variant, the H1 antigen (fliC) to be r, and the H2 antigen (fljB) to be z_53_. The strain was tentatively classified as *Salmonella enterica* subsp. *diarizonae* (subspecies IIIb) serotype 58:r:z_53_ and named *Salmonella* IIIb 58:r:z_53_.

### 2.4. Detection and Initial Characterization of S. diarizonae 58:r:z_53_ Plasmids

**Figure 3:**
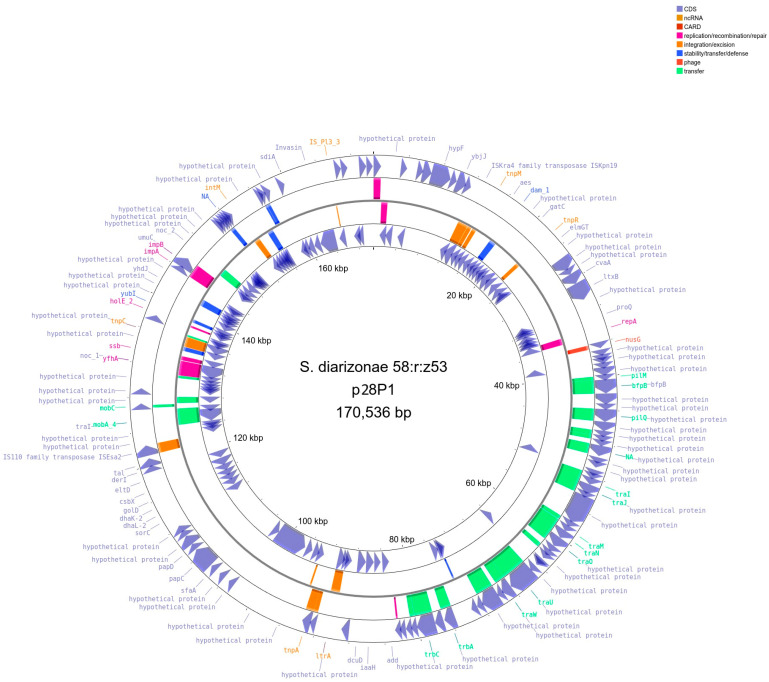
Illustration of the *S*. *diarizonae* 58:r:z_53_ plasmid 1 (p28P1, length 170,536 base pairs (bp)). The outermost and innermost rings represent the coding orientation, with the forward and reverse strands, respectively. The two central rings present mobile genetic element (MGE) annotation with the mobile orthologous groups database (Mobile OG DB). Regions involved in stability/transfer/defense are shown in blue, plasmid transfer in green, integration/excision in orange, and replication/recombination/repair in pink. Created with Proksee [17].

**Figure 4:**
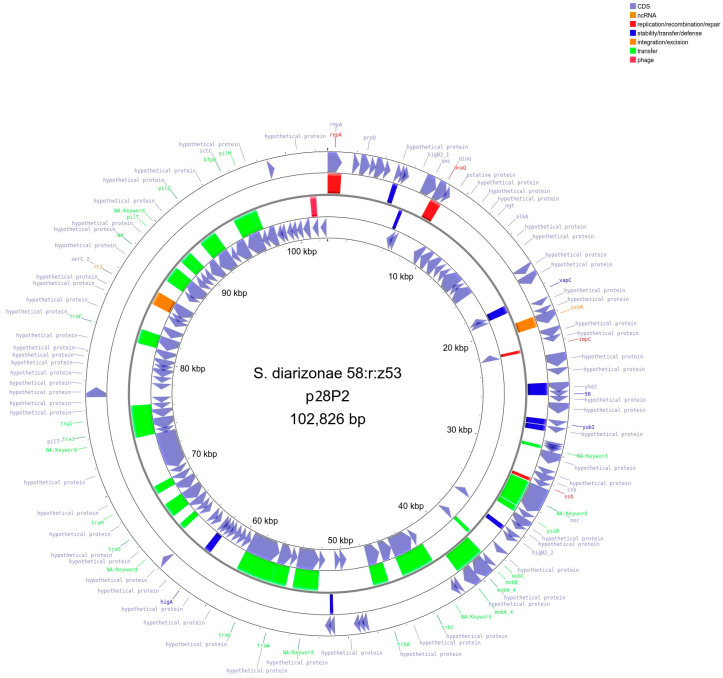
Illustration of *S*. *diarizonae* 58:r:z_53_ plasmid 2 (p28P2, length 102,826 bp). The outermost and innermost rings represent the coding orientation, with the forward and reverse strands, respectively. The two central rings present MGE annotation with Mobile OG DB. Regions involved in stability/transfer/defense are shown in blue, plasmid transfer in green, integration/excision in orange, and replication/recombination/repair in pink. Created with Proksee [17].

**Figure 5:**
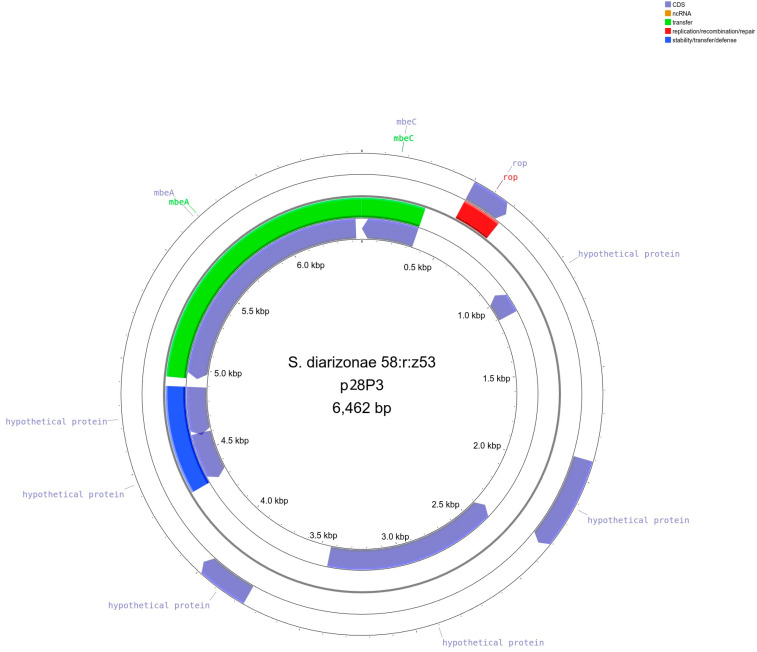
Illustration of *S*. *diarizonae* 58:r:z_53_ plasmid 3 (p28P3, 6462 bp). The outermost and innermost rings represent the coding orientation, with the forward and reverse strands, respectively. Regions involved in stability/transfer/defense are shown in blue, plasmid transfer in green, and replication/recombination/repair in pink. Created with Proksee [17].

Genome sequencing revealed that *S*. *diarizonae* 58:r:z_53_ contained three circular plasmids: plasmid 1 (p28P1, length 170,536 bp, Figure 3), plasmid 2 (p28P2, length 102,826 bp, Figure 4), and plasmid 3 (p28P3, 6462 bp, Figure 5). The plasmids did not carry antibiotic resistance genes but encoded several hypothetical proteins of unknown function, putative regulatory proteins, and genes associated with conjugation.

### 2.5. Salmonella diarizonae 58:r:z_53_ Pathogenicity Islands

*Salmonella* IIIb 58:r:z_53_ harbored six known pathogenicity islands (SPI-1, SPI-2, SPI-3, SPI-5, SPI-9, and SPI-13). An iron transport system was detected in SPI-1 centisome C63PI (Table 1).

### 2.6. Virulence and Pathogenic Genes

The chromosome of *S*. *diarizonae* 58:r:z_53_ carried multiple virulence genes including type-III secretion systems, i.e., *invA-J*, *spaO-S*, *sipA-D*, *sptP*, *prgH-J*, *orgA-C*, *avrA*, cytolethal distending toxin B (*cdtB*), *sopB*, *sopD2*, *sopE2*, *steC*, *sseF, sseG*, *sseJ*, *ssaC*, *ssaD*, *ssaG-V*, *sseB-D*, and *slrP.* Besides the type-III secretion system genes, others for virulence in the chromosome were those for magnesium uptake (*mgtA* and *mgtC*), invasion proteins (*orgA*, *orgB*, and *orgC*), iron transporters (*sitA*, *sitB*, *sitC*, and *sitD*), and aerobactin siderophores (*iucA*, iucB, and *iucC*). Yersiniabactin genes involved in iron uptake (*ybtA*, *ybtE*, *ybtP*, *ybtQ*, *ybtT*, *ybtU*, *ybtX*, *ybt S*, and *irp1,2*) were also carried. The *S*. *diarizonae* 58:r:z_53_ genome was observed to contain operons of the chaperone/usher assembly class fimbrial genes: *stc* (*yehB* and *yehB*), *bcf* (*bcfF*), *fim* (*fimA*, *fimC*, *fimD*, *fimF*, *fimH*, *fimI*, *fimW*, *fimY*, and *fimZ*), *stb* (*stbB*, *stbC*, and *stbD*), and *std* (*stdA*, *stdB*, and *stdC*), all located on the chromosome. Genes coding thin aggregative fimbria (*csgA*, *csgB*, *csgC*, *csgD*, *csgE*, *csgF*, and *csgG*) and antimicrobial peptide resistance protein Mig-14 were also components of the genome. Plasmids p28P1 and p28P2 bore a significant portion of the SPI-7 genes predicted to be related to virulence (the *pil*-locus and *tra*-region). The *relE* and *vapC* genes were also identified in plasmid p28P2.

### 2.7. Comparative Analysis of Virulence Determinants in Salmonella enterica subsp. diarizonae Strains

**Figure 6:**
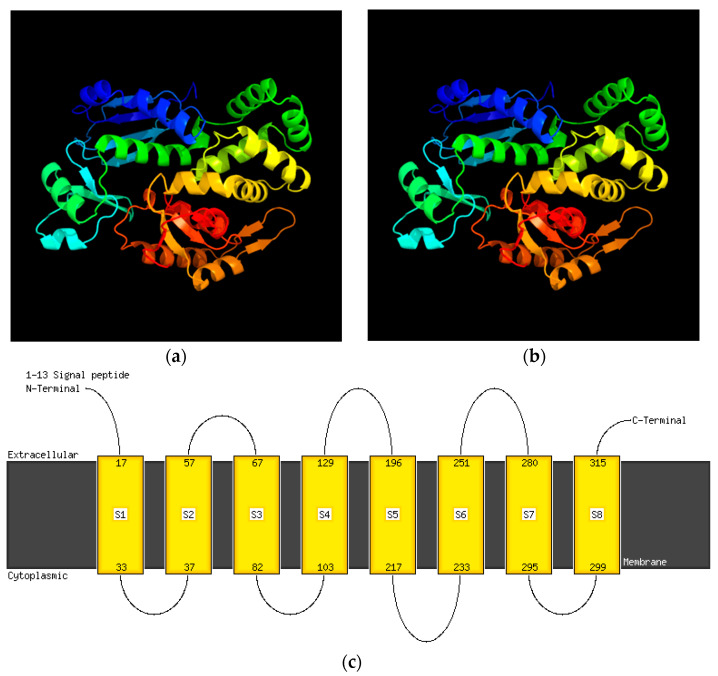
Three-dimensional view of (**a**) the full-length InvA protein (685 amino acids (aa)), (**b**) the truncated *S*. *diarizonae* 58:r:z_53_ InvA protein (665 aa), and (**c**) the truncated *S. diarizonae* 58:r:z_53_ InvA protein with no loss of transmembrane structure. Created with Phyre2, Protein Homology/analogY Recognition Engine V 2.0, Structural Bioinformatics Group, Imperial College, London, UK. (**a**,**b**) are rainbow-colored from the N to C terminus.

*Salmonella diarizonae* 58:r:z_53_ InvA invasion protein was truncated (665 amino acids (aa)) and lacked the first 20 aa present in clinical isolates of *Salmonella diarizonae* (GenBank accession numbers CP059886.1, CP123007.1, CP054422.1, and CP011288.1) [18]. This truncation did not result in the loss of the InvA transmembrane structure (Figure 6, Supplementary Figure S1).

**Figure 7:**
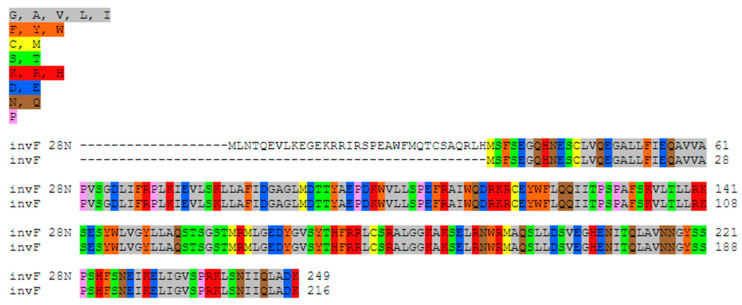
Alignment of the InvF 28N protein isolated from *S*. *diarizonae* 58:r:z_53_ (249 aa) and the same protein from a clinical strain (GenBank accession number CP011288.1, 216 aa). Created with Color Align Properties in the Sequence Manipulation Suite (https://www.bioinformatics.org/sms2/color_align_prop.html, accessed on 26 April 2024).

**Figure 8:**
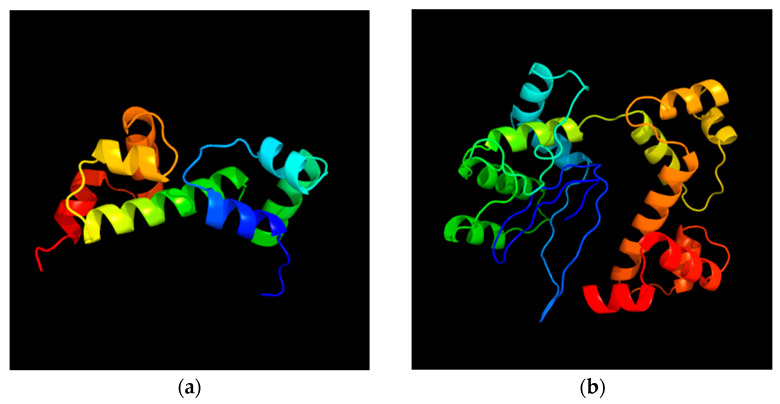
Three-dimensional view of (**a**) the truncated InvF protein (216 aa, 24.34 kDa) in *S. diarizonae* clinical isolates and (**b**) the full-length *S*. *diarizonae* 58:r:z_53_ 28N InvF protein (249 aa, 28.30 kDa). Created by Phyre2. (**a**,**b**) are rainbow-colored from the N to C terminus.

In contrast, the InvF protein was truncated (216 aa) in the clinical strains referred to in [18,19], one of which caused diarrhea and sepsis with a fatal outcome (GenBank accession number CP011288.1) [19] but these proteins had 100% identity with the InvF protein of the isolate (249 aa) (Figure 7 and Figure 8, secondary structure Figures S2 and S3) [20]. The fimbrial biogenesis chaperone StbB protein of *S*. *diarizonae* 58:r:z_53_ shared 100% of its sequence (253 aa) with that of an *S*. Typhi serovar (NCBI RefSeq number WP_053508616.1) and the fimbrial outer membrane usher StbC protein also shared its full sequence (230 aa) with that of *Salmonella enterica* subsp. *diarizonae* isolated from children (GenBank accession number JAHQRS010000001.1) [11,21].

Other invasion proteins (InfE and InvG), SPI-1 type-III secretion-system export apparatus proteins (SpaP, SpaQ, and SpaS), SPI-2 type-III secretion-system apparatus proteins (SsaS, SsaR, SsaP, and SsaK), the EscJ/YscJ/HrcJ family type-III secretion inner-membrane ring protein (SsaJ), the type-III secretion-system needle filament protein (SsaG), and the pathogenicity island 2 effector protein (SseG) showed 100% identity with their equivalents in clinical isolates of *Salmonella diarizonae* (GenBank accession numbers CP059886.1, CP123007.1, CP054422.1, and CP011288.1)

### 2.8. Antimicrobial Resistance of Salmonella IIIb 58:r:z_53_

A total of 20 different antimicrobial resistance genes were identified, all having been detected in the chromosome. Additionally, the *tolC*, *marA*, *sdiA*, and *emrD* genes were detected, which are associated with resistance to disinfecting agents and antiseptics including triclosan (Table 2, Supplementary Table S1). 

The concordance between phenotypic resistance and the presence of known AMR genes was not consistent with the genotype for all antimicrobials. Most of the detected AMR genes were associated with aminoglycoside resistance and were in a *Salmonella* isolate, which was phenotypically gentamycin-, streptomycin-, and neomycin-resistant. Similarly, the present macrolide-resistance genes were associated with phenotypic resistance to erythromycin and tylosin in the IIIb 58:r:z_53_ strain. Fluoroquinolone-resistance genes were present in the *S*. IIIb 58:r:z_53_ strain, which was nevertheless sensitive to enrofloxacin and marbofloxacin but not to flumequine. *Salmonella* IIIb 58:r:z_53_ showed intermediate resistance to florphenicol and four genes (*emrD*, *sdiA*, *acrA*, and *acrB*) from the multidrug efflux pump family were identified. The carriage of *tolC* and *marA*, which confer carbapenem resistance, was detected in *Salmonella* IIIb 58:r:z_53_ but did not prevent the strain from being sensitive to imipenem.

### 2.9. Phylogenetic Analysis of Salmonella IIIb 58:r:z_53_

To infer the phylogenetic affinities of the *Salmonella* IIIb 58:r:z_53_ isolate, the maximum-likelihood phylogeny was estimated using kSNP4 (https://sourceforge.net/projects/ksnp/) and based on the core single-nucleotide polymorphisms computed from 31 genomes downloaded from the GenBank database (Supplementary Table S2). Additionally, whole-genome- and whole-proteome-based trees were inferred using the TYGS strain-typing server (Supplementary Figure S4).

**Figure 9:**
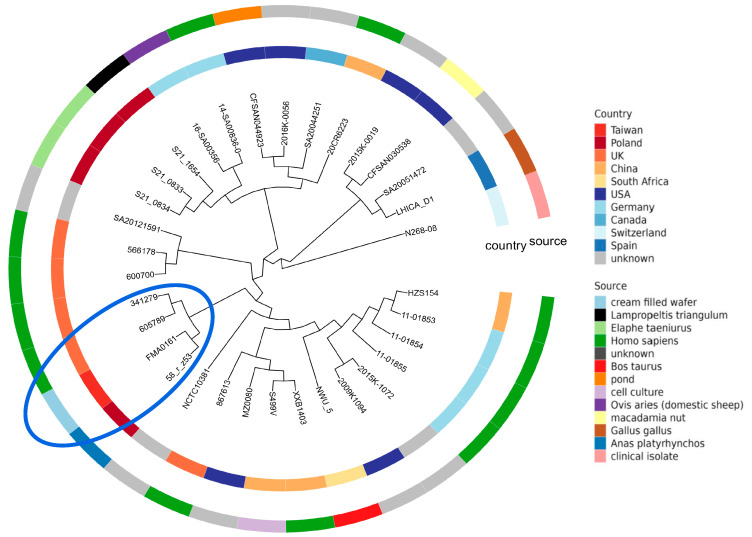
Phylogenetic analysis of the *Salmonella enterica* subsp. *diarizonae* IIIb 58:r:z_53_ strain as a maximum-likelihood tree of *S*. *diarizonae* isolates built using core genome single-nucleotide polymorphisms. The inner ring represents the country from which each isolate originated and the outer ring indicates the isolation source. Closely related strains are marked by the blue oval. *Salmonella bongori* N268-08 was used as the outgroup. All strains are presented in Table S2. Single-nucleotide polymorphisms were identified with kSNP4 [22].

Phylogenetic analysis using both tools showed that the strains named in the National Center for Biotechnology Information BioSample database as *Salmonella enterica diarizonae* serovar *Salmonella arizonae* 341279 and *Salmonella enterica diarizonae* serovar *Salmonella diarizonae* 605789 isolated from humans in the UK formed a single distinct clade with the tested strain. This single clade also included strain FMA0161 (*Salmonella enterica* subsp. *diarizonae* IIIb 58:r:-) isolated from cream-filled wafers in Taiwan in 2011, which showed the highest average nucleotide identity (99.65%) and conserved DNA threshold (84.15%) (Figure 9) (Supplementary Table S2).

## 3. Discussion

*Salmonella enterica* subsp. *enterica* is responsible for over 99% of cases of human salmonellosis and is therefore the subject of extensive research. However, there is still only very limited published research and genomic information about the non-enterica subspecies [12,13,23,24]. Our whole-genome analysis revealed that there were six known *Salmonella* pathogenicity islands in *Salmonella* IIIb 58:r:z_53_ (SPI-1, SPI-2, SPI-3, SPI-5, SPI-9, and SPI-13) and there was also centisome C63PI, an iron transport system. Genes from SPI-2, SPI-3, and SPI-13 are reported to be required for *S*. Typhimurium survival and replication in macrophages [24]. All of them were found in *Salmonella* IIIb 58:r:z_53_. In addition, SPI-1 and SPI-5, which are crucial for the intracellular lifestyle of the pathogen, were present in *Salmonella* IIIb 58:r:z_53_. The iron transport system C63PI in SPI-1, crucial for the entry of *Salmonella* into host cells, was also found in *Salmonella* IIIb 58:r:z_53_ [25].

Plasmids P1 and P2 contained many of the SPI-7 genes predicted to be related to virulence (the type IVb pilus *pil* and *tra* regions). Therefore, the presence of the *pil* locus in diarrheagenic bacteria may have contributed to their ability to infect humans.

The comparison of *Salmonella* IIIb 58:r:z_53_ with *S. diarizonae* isolated from newborn child infections revealed that 11 out of 12 type-III effector genes were present in all strains, namely *steC*, *sseJ*, *sseG*, *sseF*, *sptP*, *sopE2*, *sopB*, *sipA*, *sipB*, *sipD*, and *avrA* [26]. Moreover, *Salmonella* IIIb 58:r:z_53_ harbored the gene encoding the glycosyltransferase SseK2, which interferes with the proper immune response to infection through tumor necrosis factor-alpha-stimulated nuclear factor kappa B cell signaling. The host immune response is also hampered by the E3 ubiquitin ligase SlrP, known to have been produced by *S. diarizonae* and isolated from human and animal infections and by *Salmonella* IIIb 58:r:z_53_ because it inhibits the release of interleukin-1β. Fimbriae on the *Salmonella* cell surface mediate adhesion to host cells [27]. *Salmonella* IIIb 58:r:z_53_ bore the *mig-14* gene coding antimicrobial resistance protein Mig-14, important in bacterial resistance to antimicrobial peptides and necessary for replication of the *S.* Typhimurium serovar in the liver and spleen [28].

The *Salmonella* IIIb 58:r:z_53_ P1 plasmid bore the *samA* gene, part of the *samAB* operon. The operon of which *samA* is part efficiently promotes UV mutagenesis when carried on a high-copy-number 60-MDa cryptic plasmid, as observed in research concerning the *samAB* operon in the Typhimurium serovar chromosome [29].

Aminoglycosides interfere with bacterial protein synthesis by binding to the bacterial 30S or ribosomal subunit. Aminoglycoside 6ʹ-N-acetyltransferase (AAC(6ʹ)) inactivates aminoglycoside antibiotics through acetylation of the 6-amino-acid group of the compound. One aminoglycoside found in *S. enterica*, AAC(6′)-Iy, is a cryptic chromosomally encoded aminoglycoside acetyltransferase that has been shown to confer extensive aminoglycoside resistance in strains expressing the structural gene.

Most of the *Salmonella* IIIb 58:r:z_53_ AMR mechanism was associated with its antibiotic efflux pump. Genes involved in the pump mechanism—*marR* encoding the MarR regulator of the AcrAB multidrug efflux pump and *msbA* encoding the multidrug resistance transporter—were found in *Salmonella* IIIb 58:r:z_53_ and *S*. *diarizonae* isolated from invasive newborn child infections [26]. Additionally, the *Salmonella* IIIb 58:r:z_53_ genome also contained *tolC*, *marA*, *sdiA*, and *emrD* associated with resistance to disinfecting agents and antiseptics including triclosan. A human isolate of *S. enterica* subsp. *diarizonae* serovar IIIb 48:i:z was found to contain the *marA* gene [26].

Three approaches to phylogenetic relationship reconstruction were taken in this study: core SNP identification and whole-genome- and whole-proteome-based strategies. They revealed that the *S*. IIIb 58:r:z_53_ strain was clustered with a Taiwanese strain (isolated from food) and *S. enterica* subsp. *diarizonae* from the UK (isolated from humans). This shows that the isolate presented in this study could unquestionably infect humans and the fact that it could be present in meat for human consumption indicates that it could be widespread and a real threat to public health.

## 4. Materials and Methods

### 4.1. Sampled Animal

The analyzed *S*. *enterica* spp. *diarizonae* (*S.* IIIb 58:r:z_53_) strain was isolated from the intestine of a mallard duck (*Anas platyrhynchos*) shot as prey in accordance with the hunting law in force in Poland [30,31]. The mallard duck was in good physical condition, with no pathological changes observed when given an anatomopathological examination.

### 4.2. Salmonella spp. Isolation and Identification

*Salmonella* spp. were isolated in accordance with PN-EN ISO 6579-1:2017-04 Microbiology of the food chain—horizontal method for the detection, enumeration, and serotyping of *Salmonella*—Part 1: Detection of *Salmonella* spp. [32]. The microbiological media used were described in a publication by Pławińska-Czarnak et al. [15]. Biochemical strain identification was performed using a VITEK^®^ 2 GN (Gram-Negative) card and API20E test (BioMérieux, Craponne, France) according to the manufacturer’s instructions. For serological typing, the strains were originally grown on 2% nutrient agar slants and re-isolated on Salmonella-Shigella agar, Hektoen agar, Bismuth sulfite agar, and Xylose Lysine Deoxycholate agar (Merck, Darmstadt, Germany) before serotyping. The presumptive colonies of *Salmonella* strains were chosen and were cultured on Enrichment LAB-AGAR^TM^ (BioMaxima, Lublin, Poland) at 37 °C overnight. These cultures were used for serological identification. The antigenic formula of the *Salmonella* strains was determined with the use of standard agglutination methods and the serotype name was assigned according to the White–Kaufmann–LeMinor (WKL) scheme [1,33]. A small amount of bacterial mass from one colony was first checked by slide agglutination for a positive reaction with polyvalent HM serum and next, somatic O antigen was identified by slide agglutination in a drop of serum. Then, each strain was grown on swarm agar plates (BioMaxima, Lublin, Poland) at 37 °C overnight to test phases 1 and 2 of H antigens by slide agglutination. Polyvalent and monovalent anti-O and anti-H diagnostic sera for *Salmonella* antigens were purchased from SSI Diagnostica A/S (Hillerød, Denmark), Sifin Diagnostics GmbH (Berlin, Germany), and BIOMED (Kraków, Poland). *Salmonella* antigens were classified into serotypes by the WKL scheme. When a serovar had not been previously recorded in Poland, the strain representing this newly recognized serovar was sent to the WHO Collaborating Centre for Reference and Research on *Salmonella* (Institut Pasteur, Paris, France) for the identification to be verified and confirmed [15].

### 4.3. Antimicrobial Sensitivity Testing

The *Salmonella* strain was subcultured as described previously. From an 18–24 h culture, a suspension was prepared to 0.5 McFarland turbidity with a DensiCHEK Plus instrument (BioMérieux, Marcy-l’Étoile, France) and the inoculum was transferred to another VITEK^®^ tube containing 3 mL of 0.45% saline. The card was automatically filled by a vacuum device and automatically sealed. It was manually inserted in the VITEK2 Compact reader–incubator module (BioMérieux, Craponne, France) and the card was automatically subjected to a kinetic fluorescence measurement every 15 min. This is a test methodology based on the minimum inhibitory concentration (MIC) technique reported by MacLowry and Marsh [34] and Gerlach [35]. To analyze MIC patterns of *S. enterica* subsp. *diarizonae*, a MERLIN MICRONAUT system (MERLIN Diagnostika GmbH, Bremen, Germany) was used. The MICs were interpreted according to the Clinical and Laboratory Standards Institute and Food and Drug Administration breakpoints [36]. The antimicrobial susceptibility was assessed by determining the MIC values using 96-well MICRONAUT Special Plates in a protocol described by Pławińska-Czarnak et al. in 2022 [37].

### 4.4. Whole-Genome Sequencing

Whole-genome sequencing of *Salmonella* spp. was performed at Genomed S.A. (Warsaw, Poland). Illumina sequencing was conducted in paired-end 300 bp mode on the MiSeq device (Illumina, San Diego, CA, USA) targeting 100× genome coverage. Sequencing was also performed using an SQK-RBK004 kit and an R9.4.1 flow cell on a MinION instrument (Oxford Nanopore Technologies, Oxford, UK). Short-read quality was assessed using FASTQC (https://www.bioinformatics.babraham.ac.uk/projects/fastqc) and sequencing data were quality trimmed using fastp [38]. Long reads were quality-filtered using NanoFilt [39] and the residual adaptor was removed using Porechop (https://github.com/rrwick/Porechop, accessed on 3 February 2024). The filtered dataset was finally quality-checked using NanoPlot [39]. Long-read assembly was performed using the Trycycler pipeline [40]. In brief, nanopore reads were initially assembled using four long-read assemblers—flye v. 2.9, unicycler v. 04.8, Raven v. 1.8.1, and miniasm v. 0.3-r179—next, the assemblies were reconciled and circularized, the final consensus was generated, and it was polished with Medaka (Oxford Nanopore Technologies). Circular replicons were further polished with short Illumina reads using the Polypolish (https://github.com/rrwick/Polypolish) [40] and POLCA [41] programs. The remaining ambiguities in the genome assembly were verified by PCR amplification of DNA fragments followed by Sanger sequencing with an ABI3730xl Genetic Analyzer (Life Technologies, Carlsbad, CA, USA) using BigDye Terminator Mix v. 3.1 chemistry (Life Technologies). All of the possible sequence errors and misassemblies were further manually corrected using Seqman software (DNAStar, Madison, WI, USA, https://www.dnastar.com/software/lasergene/seqman-ultra/?utm_source=google&utm_medium=cpc&utm_campaign=6773110442&utm_content=134971714457&utm_term=&utm_term=&utm_campaign=Branded+Keywords&utm_source=adwords&utm_medium=ppc&hsa_acc=7537872482&hsa_cam=6773) to obtain the complete nucleotide sequence of the bacterial genome.

### 4.5. Whole-Genome Analysis of Salmonella enterica spp. diarizonae 58:r:z_53_

General information about the assembly quality and gene content of *Salmonella enterica* subsp. *diarizonae* 58:r:z_53_ isolates and genomic components were obtained using the genomics tools of the Bacterial and Viral Bioinformatics Resource Center (BV-BRC, https://www.bv-brc.org, accessed on 29 February 2024). The serotype of strain *S*. IIIb 58:r:z_53_ was elucidated using SeqSero2 [42,43] and the genome sequence was annotated using Bakta software v. 1.8.2 and the Bakta database v. 5.0 (https://github.com/oschwengers/bakta) [44]. *Salmonella* pathogenic islands were detected using SPIFinder (https://cge.food.dtu.dk/services/SPIFinder/, accessed on 29 February 2024). Antibiotic resistance and virulence factor coding genes were detected using abricate (https://github.com/tseemann/abricate) by screening against the CARD (https://card.mcmaster.ca/) and VFDB (http://www.mgc.ac.cn/VFs/) databases, respectively. Genome maps of the *S.* IIIb 58:r:z_53_-strain chromosome and plasmids were visualized using the Proksee [17] and GenoVi packages [16].

### 4.6. Phylogenetic Analysis

The phylogenetic analysis of *S*. IIIb strain 58:r:z_53_ was performed including the genomic sequences of other *S. enterica* subsp. *diarizonae* members that are available in GenBank.

In total, 31 sequences were selected using the Referenceseeker tool [45] and based on an ANI value of 99% and conserved DNA threshold of 0.69. Those 31 comprised 10 incomplete genome sequences (drafts) and 21 complete ones. *Salmonella bongori* N268-08 was used as the outgroup. The sequences were uploaded to the TYGS typing server [46] and a whole-genome sequence-based tree was constructed.

Phylogenetic analysis was also performed taking a reference-free SNP-based approach using kSNP4 software [22]. A phylogenetic tree was constructed based on the maximum-likelihood method and visualized using the ggtree R package 3.12.0 (https://bioconductor.org/packages/release/bioc/html/ggtree.html, accessed on 29 February 2024).

The Phyre2 [47] web portal served as a resource for protein modeling, prediction, and analysis (http://www.sbg.bio.ic.ac.uk/phyre2, accessed on 19 March 2024).

## Figures and Tables

**Table 1:** *Salmonella* pathogenicity islands (SPIs) detected in the genome of *Salmonella enterica* subsp. *diarizonae* 58:r:z_53_ isolated from a mallard duck in Poland and the GenBank references of previous detections of those islands.

Name	Type	Start/Stop	Identity %	Query/Template Length	Genes	Bacterium	Accession No.
C63PI	CDS	1 219 752/1 223 751	95.58	4000/4000	*sitA*, *sitB*, *sitC*, *sitD*	*Salmonella-enterica*-Typhimurium-SL1344	AF128999
SPI-1	CDS	1 190 777/1 191 191	96.14	415/415	*invA*	*Salmonella-enterica*-Gallinarum-SGB_8	AY956825
SPI-1	CDS	1 189 639/1 189 897	95.37	259/257	*invA*	*Salmonella-enterica*-Typhimurium-J4STEHO	JN982040
SPI-1	CDS	1 171 725/1 174 414	95.32	2692/3141	*fhlA/mutS*	*Salmonella-enterica*-Typhimurium-SL1344	U16303
SPI-13	CDS	745 447/745 784	98.22	338/338	*gacD*	*Salmonella-enterica*-Gallinarum-SGD_3	AY956832
SPI-13	CDS	746 092/746 495	98.02	404/404	*gtrA*	*Salmonella-enterica*-Gallinarum-SGG_1	AY956833
SPI-13	CDS	747 863/748 203	98.53	341/341	*gtrA*	*Salmonella-enterica*-Gallinarum-SGA_10	AY956834
SPI-2	CDS	3 159 479/3 164 104	95.47	4634/4631	ORF32, ORF48, *pykF*	*Salmonella-enterica*-Typhimurium-LT2	X99945
SPI-3	CDS	66 847/67 584	96.75	738/738	*mgtC*	*Salmonella-enterica*-Typhimurium-14028s	AJ000509
SPI-5	CDS	3 455 305/3 456 557	99.36	1253/1253	*sopB*	*Salmonella-diarizonae*-SARC7	AF323077
SPI-9	CDS	1 323 107/1 335 754	95.65	12651/15696		*Salmonella*-*enterica*-Typhi-CT18	NC_003198

ORF: open reading frame.

**Table 2:** Genes related to antibiotic resistance found in *S. diarizonae* 58:r:z_53_.

AMR Mechanism	Genes	Drug Class
antibiotic inactivation	*AAC(6′)-Iy*	aminoglycoside
antibiotic efflux	*acrD*, *kdpE*	aminoglycoside
	*mdtB*, *mdtC*	aminocoumarin
	*baeR*, *cpxA*	aminocoumarin, aminoglycoside
	*msbA*	nitroimidazole
	*yojI*	peptide antibiotic
	*emrA*, *emrB*, *emrR*	fluoroquinolone
	*crp*	fluoroquinolone, macrolide, penam
	*msrB*	streptogramin, macrolide
	*h-ns*	macrolide, penam, cephamycin, tetracycline, cephalosporin, fluoroquinolone
	*emrD*	phenicol, disinfecting agent, antiseptic
	*sdiA*, *E. coli acrA*	cephalosporin, glycylcycline, penam, tetracycline, fluoroquinolone, rifamycin, phenicol, triclosan
	*acrB*	tetracycline, rifamycin, glycylcycline, phenicol, penam, cephalosporin, fluoroquinolone, disinfecting agent, antiseptic
	*tolC*	peptide antibiotic, aminoglycoside, tetracycline, glycylcycline, macrolide, fluoroquinolone, penam, carbapenem, penem, aminocoumarin, phenicol, cephalosporin, rifamycin, cephamycin, disinfecting agent, antiseptic
antibiotic target alteration	*bacA*	peptide antibiotic
	*glpT*, *uhpT*	fosfomycin
reduced permeability to antibiotics, antibiotic efflux	*marA*	glycylcycline, cephalosporin, penam, cephamycin, monobactam, penem, tetracycline, fluoroquinolone, rifamycin, phenicol, carbapenem, triclosan

AMR: antimicrobial resistance.

## Data Availability

The datasets presented in this study have been deposited in the BioProject database under accession number PRJNA1101854 and in the BioSample database under SAMN41003542, Genome accession numbers: CP152066-CP152069.

## Supplementary Materials

The supporting information can be downloaded at https://www.mdpi.com/article/10.3390/ijms25115664/s1.
