# Supplementary material for: Does Salmonella diarizonae 58:r:z53 Isolated from a Mallard Duck Pose a Threat to Human Health?
Source: Int J Mol Sci. 2024 May 23;25(11):5664. doi: 10.3390/ijms25115664 (PMC11171591; doi:10.3390/ijms25115664)
Supplement: Supplementary file 1 [file ijms-25-05664-s001.zip › Figure S2_Figure S3_ invF 28N (1).pdf]

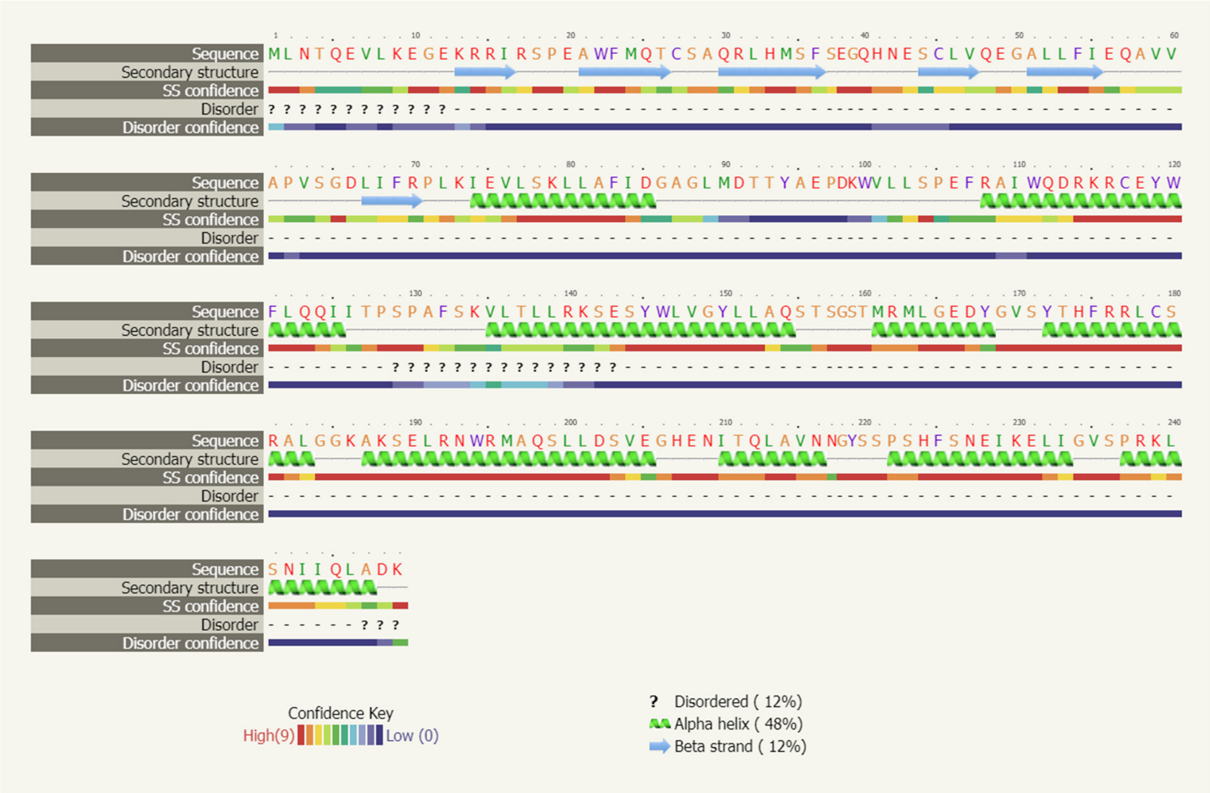

Figure S2. Secondary structure InvF (249 aa) *S. diarizonae* 58:r:z53 prediction using PHYRE2 [47]. The result shows that the majority of the core structure (48%) is alpha helix - green helix, and minority (12%) is β-strand – blue arrow, faint lines indicate coil. The confidence keys of the predicted structure for these regions are high. Disorderd line (12%) contains the prediction of disordered regions in protein.

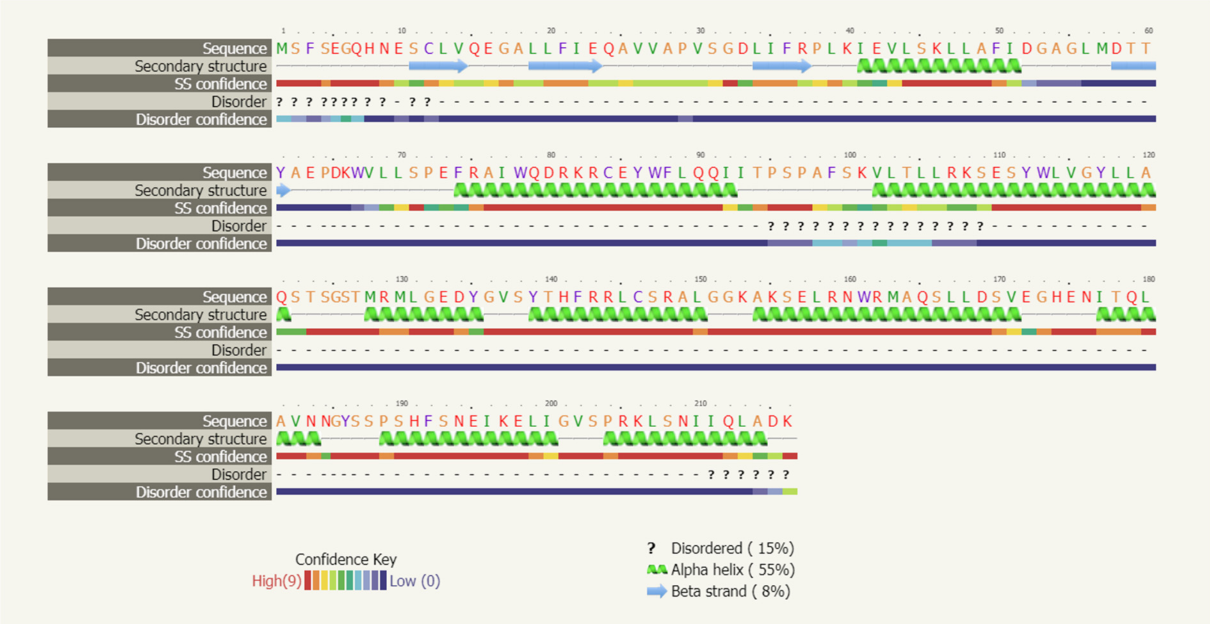

Figure S3. Secondary structure InvF (216 aa) protein present in *S. diarizonae* clinical isolates [18,19], prediction using PHYRE2. The result shows that majority of the core structure (55%)

is alpha helix - green helix, and minority (8%) is β-strand – blue arrow, faint lines indicate coil. The confidence keys of the predicted structure for these regions are high. Disorderd line (15%) contains the prediction of disordered regions in protein.
