# Supplementary material for: Does Salmonella diarizonae 58:r:z53 Isolated from a Mallard Duck Pose a Threat to Human Health?
Source: Int J Mol Sci. 2024 May 23;25(11):5664. doi: 10.3390/ijms25115664 (PMC11171591; doi:10.3390/ijms25115664)
Supplement: Supplementary file 1 [file ijms-25-05664-s001.zip › FigureS1_invA 28N (1).pdf]

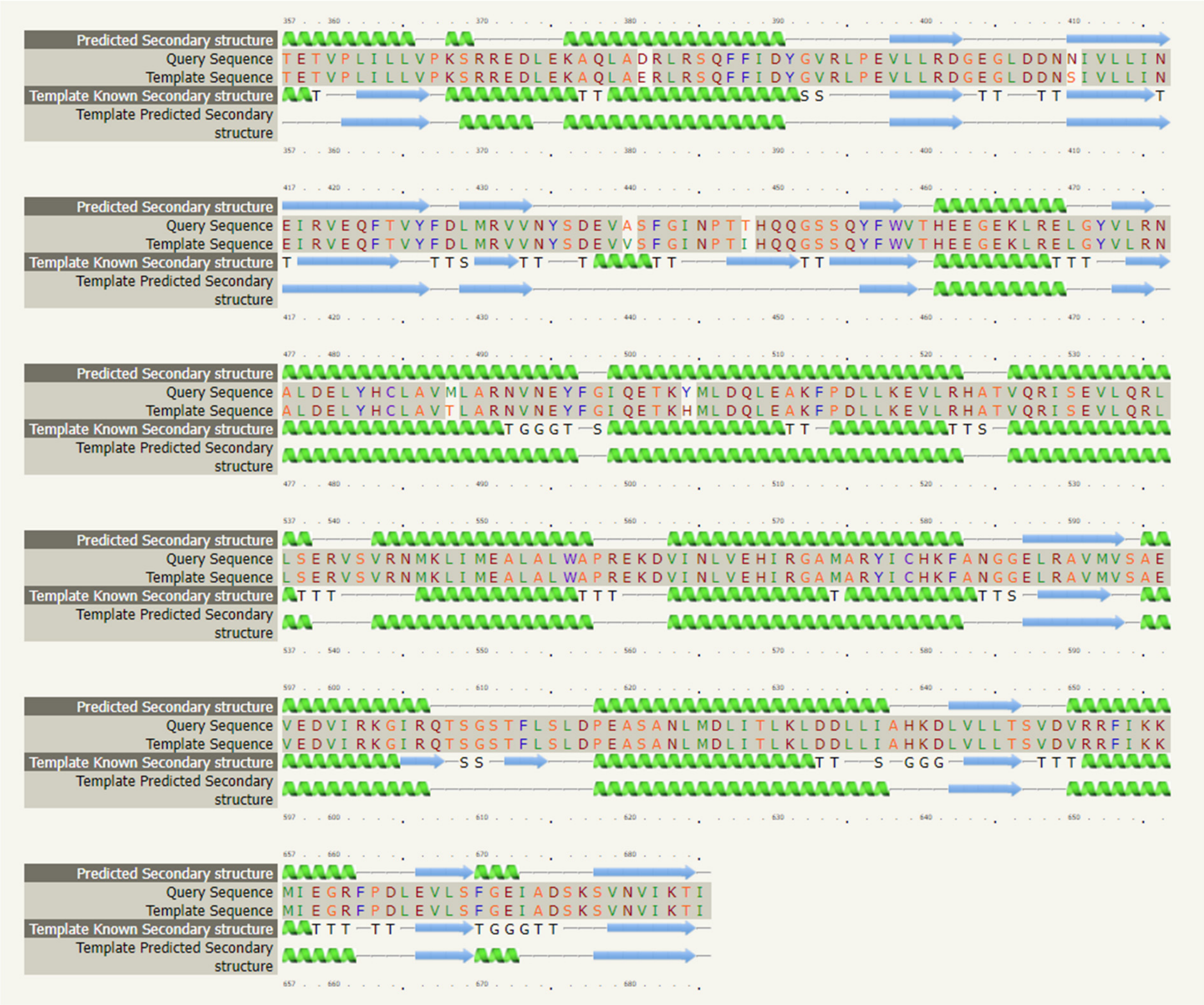

A.

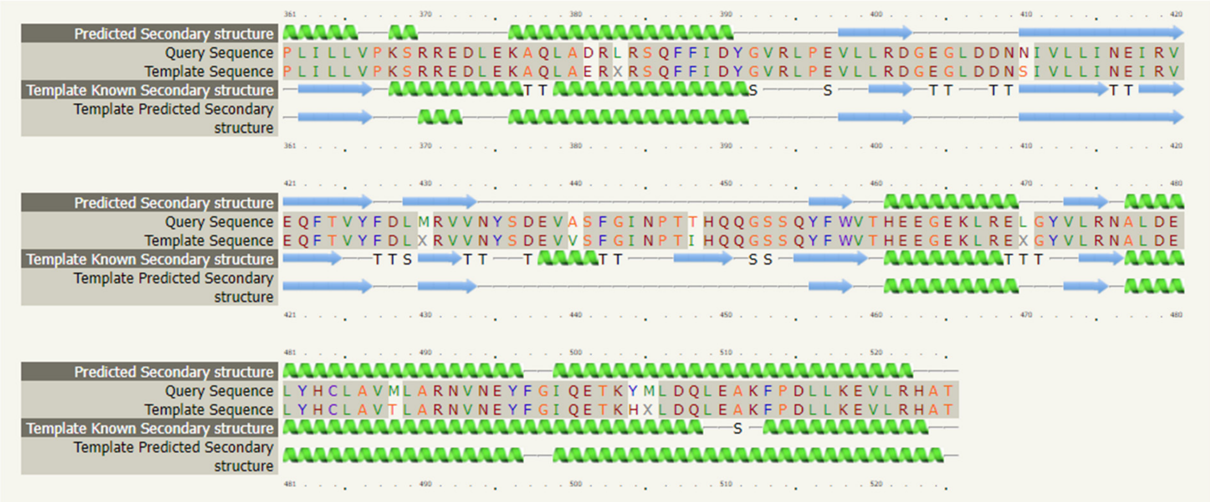

B.

C. 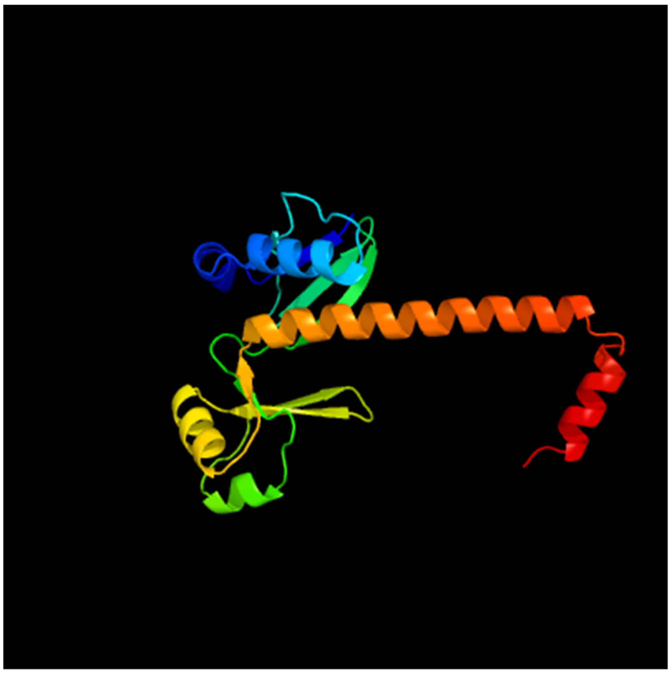

Figure S1. Alignment of the A. crystal structure of the c-terminal domain of invA protein *S. diarizonae* 58:r:z53 and *Salmonella* Typhimurium str. LT2. B. structure of a cytoplasmic domain of *Salmonella* invA *Salmonella* Typhimurium str. LT2. C. 3D viewing of the protein model structure of a cytoplasmic domain of *Salmonella IIIb 58:r:z53* invA. Created with Phyre2 [47].
