# Supplementary material for: Does Salmonella diarizonae 58:r:z53 Isolated from a Mallard Duck Pose a Threat to Human Health?
Source: Int J Mol Sci. 2024 May 23;25(11):5664. doi: 10.3390/ijms25115664 (PMC11171591; doi:10.3390/ijms25115664)
Supplement: Supplementary file 1 [file ijms-25-05664-s001.zip › Suplement_Figure_4S.pptx]

## Slide 1
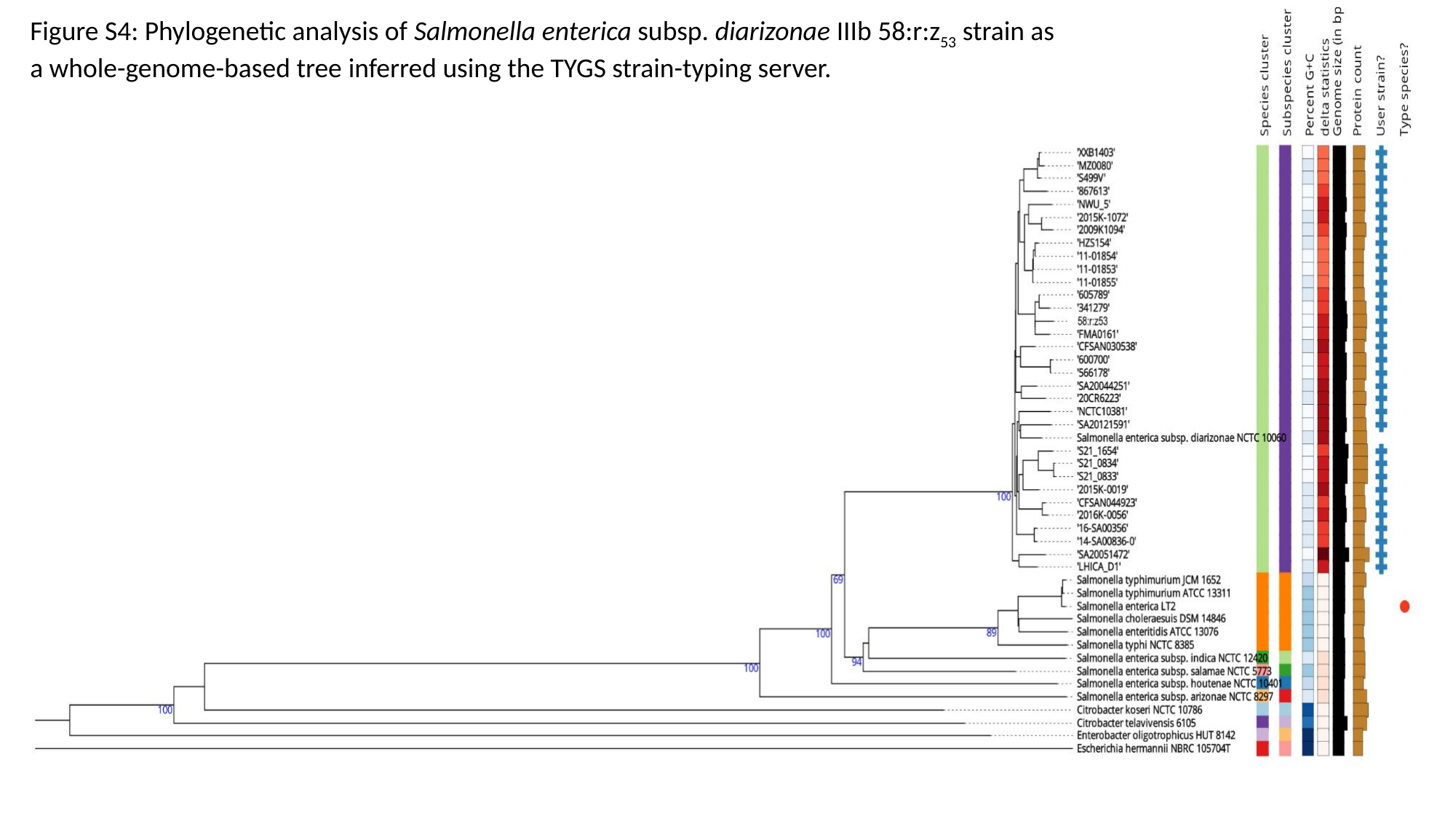

Figure S4: Phylogenetic analysis of Salmonella enterica subsp. diarizonae IIIb 58:r:z53 strain as a whole-genome-based tree inferred using the TYGS strain-typing server.

## Slide 2
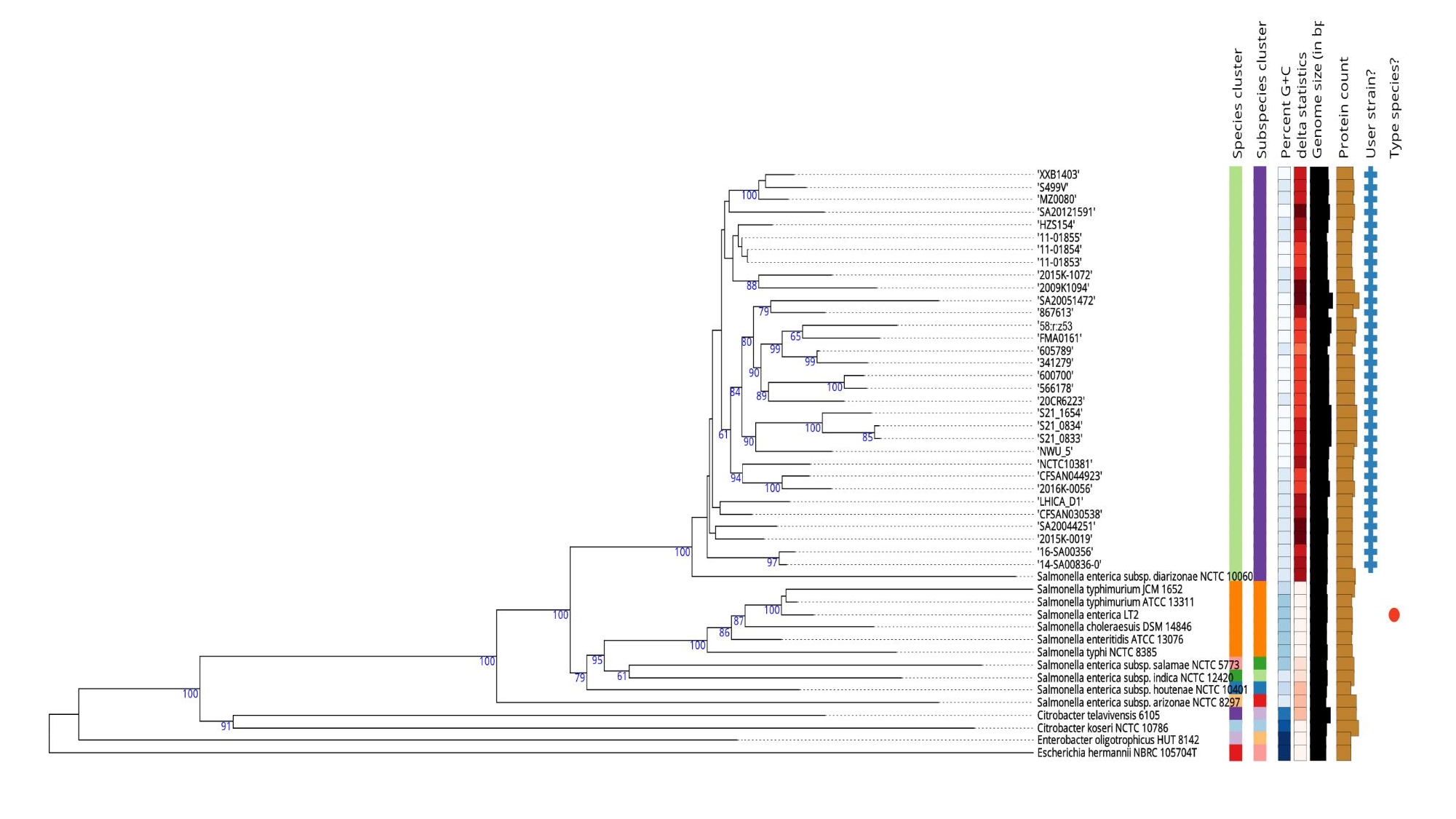

#
